# Development and external-validation of a nomogram for predicting the survival of hospitalised HIV/AIDS patients based on a large study cohort in western China

**DOI:** 10.1017/S0950268820000758

**Published:** 2020-04-01

**Authors:** Z. Yuan, B. Zhou, S. Meng, J. Jiang, S. Huang, X. Lu, N. Wu, Z. Xie, J. Deng, X. Chen, J. Liu, J. Zhang, F. Wu, H. Liang, L. Ye

**Affiliations:** 1 Guangxi Key Laboratory of AIDS Prevention and Treatment, School of Public Health, Guangxi Medical University, Nanning, Guangxi, China; 2 Guangxi Collaborative Innovation Center for Biomedicine, Life Sciences Institute, Guangxi Medical University, Nanning, Guangxi, China; 3 Fourth People's Hospital of Nanning, Nanning, Guangxi, China

**Keywords:** AIDS, hospitalised patients, nomogram, survival

## Abstract

The aim of this study was to develop and externally validate a simple-to-use nomogram for predicting the survival of hospitalised human immunodeficiency virus/acquired immunodeficiency syndrome (HIV/AIDS) patients (hospitalised person living with HIV/AIDS (PLWHAs)). Hospitalised PLWHAs (*n* = 3724) between January 2012 and December 2014 were enrolled in the training cohort. HIV-infected inpatients (*n* = 1987) admitted in 2015 were included as the external-validation cohort. The least absolute shrinkage and selection operator method was used to perform data dimension reduction and select the optimal predictors. The nomogram incorporated 11 independent predictors, including occupation, antiretroviral therapy, pneumonia, tuberculosis, *Talaromyces marneffei*, hypertension, septicemia, anaemia, respiratory failure, hypoproteinemia and electrolyte disturbances. The Likelihood χ^2^ statistic of the model was 516.30 (*P* = 0.000). Integrated Brier Score was 0.076 and Brier scores of the nomogram at the 10-day and 20-day time points were 0.046 and 0.071, respectively. The area under the curves for receiver operating characteristic were 0.819 and 0.828, and precision-recall curves were 0.242 and 0.378 at two time points. Calibration plots and decision curve analysis in the two sets showed good performance and a high net benefit of nomogram. In conclusion, the nomogram developed in the current study has relatively high calibration and is clinically useful. It provides a convenient and useful tool for timely clinical decision-making and the risk management of hospitalised PLWHAs.

## Introduction

Acquired immunodeficiency syndrome (AIDS) caused by human immunodeficiency virus (HIV) infection is still a major global public health issue. Globally, in 2017, nearly 36.9 million people lived with HIV/AIDS and 940 000 people died from AIDS-related illnesses [1]. In recent years, the mortality of HIV-infected individuals in China has dramatically declined with the introduction of antiretroviral therapy (ART) [2]. However, the hospital inpatient mortality among HIV-infected patients in China remained at a high level even in the ART era [3, 4].

HIV infection disrupts the host immune system, resulting in high risks for various comorbidities, including opportunistic infections (OIs), malignancies and other consequences of immunodeficiency. The common HIV-related OIs include tuberculosis, pneumocystis pneumonia, Candida and herpesvirus [5]. Two earlier studies in China explored the risks of inpatients infected with HIV and showed that OIs may account for most of the deaths [2, 6]. In addition to OIs, malignancies [7] (e.g., Kaposi's sarcoma, B cell lymphoma and non-Hodgkin's lymphoma) and other comorbidities [8, 9] (e.g., pulmonary arterial hypertension, malnutrition and emphysema) may also exacerbate the disease progression and result in the death of inpatients with HIV/AIDS.

Hospitalised person living with HIV/AIDS (PLWHAs) have a variety of HIV-related OIs with different climates and socio-economic conditions [3, 10]. Guangxi, an economically less-developed province in southern China, borders Vietnam and has a subtropical monsoon climate. Guangxi owns the Beibu Gulf Economic Zone, with a large cross-border floating population. A large number of HIV-infected sex workers and drug users also gather in this area [11, 12]. Currently, Guangxi has the second-highest reported cases of HIV infections in China. HIV/AIDS patients in Guangxi more often suffer from various comorbidities and have much higher mortality than the national average level [13, 14]. For example, in Guangxi, approximately 16.1% of hospitalised HIV-infected patients were coinfected with *Talaromyces marneffei,* which is rarely found in most other areas of China and accounts for 17.5% of all deaths among HIV-infected inpatients in Guangxi [15].

To improve the life expectancy and quality of life of hospitalised PLWHAs, adopting individualised treatment programs in a timely manner is the most preferred strategy. This requires clinicians to be able to assess the condition of patients earlier based on admission examination information. Therefore, a simple-to-use tool that fulfils doctors' needs is necessary. Medical nomograms are a pictorial representation for clinical usage that integrates biological and clinical data, such as age, sex, complications and biochemical test results. Nomograms have been proposed to predict survival and prognosis in oncology and medicine [16, 17]. In oncology, nomograms can incorporate patient and disease characteristics, including continuous variables, to estimate individualised risk and are superior to conventional TNM staging. In addition, nomograms can be personalised to guide the care of cancer patients. In terms of AIDS treatment, nomograms have mainly been developed for correcting and predicting the mortality of patients with HIV/AIDS after antiretroviral treatment [18] or guiding antiviral treatment strategy [19]. Recently, Joseph *et al*. [20] developed and internally validated a nomogram to evaluate the risk of HIV infection in Uganda. Nevertheless, so far, few studies have been performed to develop a simple-to-use nomogram model to predict the survival of hospitalised HIV-infected inpatients for timely individualised decision-making.

In this study, we aimed to develop and externally validate a clinic-based nomogram that incorporates the features and common comorbidities of HIV/AIDS for predicting the survival of HIV-infected patients during hospitalisation.

## Methods

### Study patients and data collection

**Fig. 1.**
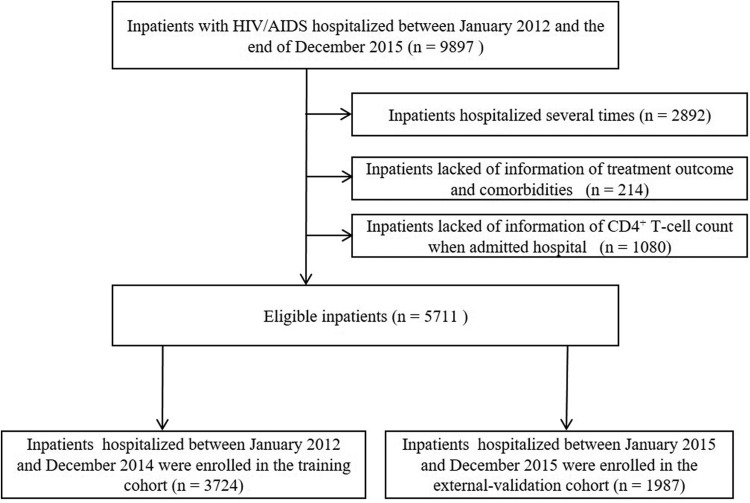
Selection process of subjects for the construction of the nomogram.

A large-scale retrospective cohort study was conducted in the Fourth People's Hospital of Nanning (Guangxi, China) as we reported previously [15]. The Fourth People's Hospital of Nanning (Guangxi, China) is a designated hospital for AIDS treatment in Guangxi. It is also the largest infectious disease prevention and treatment hospital in Guangxi mainly for the treatment of AIDS and opportunistic infections. Hospitalised PLWHAs recorded in the hospital electronic medical record system between January 2012 and December 2015 were included in this study. The diagnosis of HIV infection was determined by ELISA and Western Blot assays. For those with multiple admissions, we collected information on the latest admission. Patients who lacked information on outcomes and comorbidities or relevant examination data (e.g., CD4^+^ T cell count) were excluded. The patients selection process is shown in Figure 1. This study was approved by the Human Research Ethics Committee of Guangxi Medical University (Ethical Review No. 2013-130) and consent was obtained from all participants (or their guardians).

The demographic characteristics, hospitalisation time or survival time in the hospital, outcomes of treatment and CD4^+^ count at admission were collected from the hospital electronic medical record system. ART history before admission was obtained from the National Free Antiretroviral Treatment Programme database. Information on the clinical diagnosis was collected from the hospital laboratory. The characteristics selected for the model were based on literature review [2, 6, 7] and advice of clinicians. Examinations for HIV-associated OIs, consequences of immunodeficiency and tumours, as well as other crucial examinations, were performed to detect the following items: immune reconstitution inflammatory syndrome (IRIS), pneumonia (excluding tuberculosis), tuberculosis, meningitis, *Talaromyces marneffei* infection, candida infection, chronic hepatitis (hepatitis B or hepatitis C), syphilis, tumour, gastritis, enteritis, dermatitis, hypertension, septicemia, anaemia, respiratory failure, diabetes, hypoproteinemia and electrolyte disturbances. To simplify the model, the age was categorised as (a) <20 years, (b) 20–40 years, (c) 41–60 years and (d) >60 years; the CD4^+^ T-cell count before hospitalisation was categorised as (a) <200 cells/μl, (b) 200–349 cells/μl and (c) ≥350 cells/μl.

### Predictor selection

In the design of the nomogram, HIV-infected inpatients admitted between January 2012 and December 2014 were included in the training cohort. The least absolute shrinkage and selection operator (LASSO) method [21] was used to perform data dimension reduction and select the optimal predictors for the nomogram. HIV-associated OIs, consequences of immunodeficiency, tumours and other crucial examinations from the training cohort were incorporated into the LASSO method. To implement the LASSO method, we adopted the ‘glmnet’ package.

### Development of the nomogram

In this study, the survival time was defined as the interval in days between the date of admission and the date of death. Survival time and variables selected by the LASSO method from the training cohort were incorporated into the Cox proportional hazards regression model. The regression coefficients and baseline survival were estimated. To test the Cox proportional hazard regression model, the Likelihood χ^2^ statistic was obtained. Then, variable axes of the nomogram were based on the regression coefficients and the survival probabilities at the 10-day and 20-day were calculated by the baseline survival. Finally, a baseline nomogram based on the training cohort was constructed with the ‘survival’, ‘foreign’ and ‘rms’ packages.

### Validation and evaluation of the nomogram

For the external validation procedure, the nomogram was subjected to a separate cohort of HIV/AIDS inpatients admitted in 2015 in the same hospital. To evaluate the performance of the nomogram, the Brier scores were calculated for the training and external validation cohorts. Brier scores can be considered as a measure of the ‘calibration’ of a set of probabilistic predictions and lower scores indicating higher predictive accuracy. Integrated Brier Score is a comprehensive measure of the predictive model at all time. Simultaneously, the receiver operating characteristic (ROC) and precision-recall (PR) curves were plotted and area under the curves (AUC) of ROC and PR were also calculated, respectively. To estimate the 95% confidence intervals (CI) of the Brier score, AUC-ROC and AUC-PR, we used the bootstrap method (1000 replications). The ‘purrr’, ‘PRROC’, ‘pROC’, ‘peperr’, ‘survcomp’ and ‘boot’ packages were used to plot and calculate above figures and parameters.

### Calibration and clinical utility of the nomogram

The calibration plots were also constructed to assess the concordance of the survival time between the predicted and actual outcomes. To assess the clinical utility of the nomogram developed in this study, the decision curve analysis (DCA) was conducted. DCA is based on the net benefits which is derived by different threshold probabilities. The DCA plot can visually display the net benefit of nomogram-assisted decisions at different threshold probabilities. On the DCA plot, in addition to the curve of the predictive model, two other lines represent two extreme assumptions (either all or no patient has the outcome). The DCA plots were drawn with the ‘rmda’ package.

### Statistical analysis

Statistical analysis was carried out using R software (version 3.5.1; http://www.R-project.org) and Statistical Package for the Social Sciences (SPSS) 23.0 (SPSS Inc., Chicago, IL, USA). The data are expressed as frequencies or percentages (%). *P* < 0.05 was considered statistically significant.

## Results

### Characteristics and clinical features of the HIV/AIDS inpatients

The characteristics and clinical features of the HIV/AIDS inpatients in the training and external validation cohorts are summarised in Table 1. A total of 5711 eligible inpatients were enrolled in this study, including 3724 in the training cohort and 1987 in the external validation cohort. A total of 336 (9.02%) inpatients in the training cohort and 149 (7.50%) inpatients in the external validation cohort died during their hospitalisation. The survival of the training cohort was 94.8% and 90.8% at the 10-day and 20-day, respectively.

**Table 1.** Characteristics and clinic features of hospitalised HIV/AIDS patients in the training cohort and external-validation cohort

Characteristics	Training cohort	External-validation cohort
	(*n* = 3724)	(*n* = 1987)
	*N* (%)	*N* (%)
Age (years)		
<20	74 (1.99)	32 (1.61)
20–40	1353 (36.33)	621 (31.25)
41–60	1497 (40.20)	784 (39.46)
>60	800 (21.48)	550 (27.68)
Sex		
Male	2699 (72.48)	1449 (72.92)
Female	1025 (27.52)	538 (27.08)
Nationality		
Han	2650 (71.16)	1213 (61.05)
Zhuang	1008 (27.07)	722 (36.34)
Other	66 (1.77)	52 (2.62)
Marital status		
Married	2400 (64.45)	1273 (64.07)
Single	599 (16.08)	350 (17.61)
Other	725 (19.47)	364 (18.32)
Occupation		
Farmer	2055 (55.18)	1056 (53.15)
Unemployed	654 (17.56)	358 (18.02)
Others	1015 (27.26)	573 (28.84)
ART before hospital admission		
Yes	2486 (66.76)	1173 (59.03)
NO	1238 (33.24)	814 (40.97)
CD4^+^ T-cell count when admitted hospital (cells/μl)		
<200	2423 (65.06)	1210 (60.90)
200–349	597 (16.03)	361 (18.17)
≥350	704 (18.90)	416 (20.94)
IRIS		
Yes	3648 (97.96)	1965 (98.89)
No	76 (2.04)	22 (1.11)
Pneumonia		
Yes	1921 (51.58)	1021 (51.38)
No	1803 (48.42)	966 (48.62)
Tuberculosis		
Yes	2248 (60.37)	1326 (66.73)
No	1476 (39.63)	661 (33.27)
Meningitis		
Yes	3632 (97.53)	1952 (98.24)
No	92 (2.47)	35 (1.76)
*Talaromyces marneffei*		
Yes	3040 (81.63)	1746 (87.87)
No	684 (18.37)	241 (12.13)
Candida		
Yes	2500 (67.13)	1415 (71.21)
No	1224 (32.87)	572 (28.79)
Hepatitis (B or C)		
Yes	3055 (82.04)	1712 (86.16)
No	669 (17.96)	275 (13.84)
Syphilis		
Yes	3598 (96.62)	1942 (97.74)
No	126 (3.38)	45 (2.26)
Tumour		
Yes	3601 (96.70)	1919 (96.58)
No	123 (3.30)	68 (3.42)
Gastritis		
Yes	3561 (95.62)	1892 (95.22)
No	163 (4.38)	95 (4.78)
Enteritis		
Yes	3553 (95.41)	1912 (96.23)
No	171 (4.59)	75 (3.77)
Dermatitis		
Yes	3532 (94.84)	1884 (94.82)
No	192 (5.16)	103 (5.18)
Hypertension		
Yes	3499 (93.96)	1871 (94.16)
No	225 (6.04)	116 (5.84)
Septicaemia		
Yes	3600 (96.67)	1937 (97.48)
No	124 (3.33)	50 (2.52)
Anaemia		
Yes	3176 (85.28)	1735 (87.32)
No	548 (14.72)	252 (12.68)
Respiratory failure		
Yes	3553 (95.41)	1921 (96.68)
No	171 (4.59)	66 (3.32)
Diabetes		
Yes	3608 (96.89)	1930 (97.13)
No	116 (3.11)	57 (2.87)
Hypoproteinemia		
Yes	3454 (92.75)	1858 (93.51)
No	270 (7.25)	129 (6.49)
Electrolyte disturbances		
Yes	3246 (87.16)	1841 (92.65)
No	478 (12.84)	146 (7.35)

ART, antiretroviral therapy; IRIS, immune reconstitution inflammatory syndrome.

### Predictor selection

**Fig. 2.**
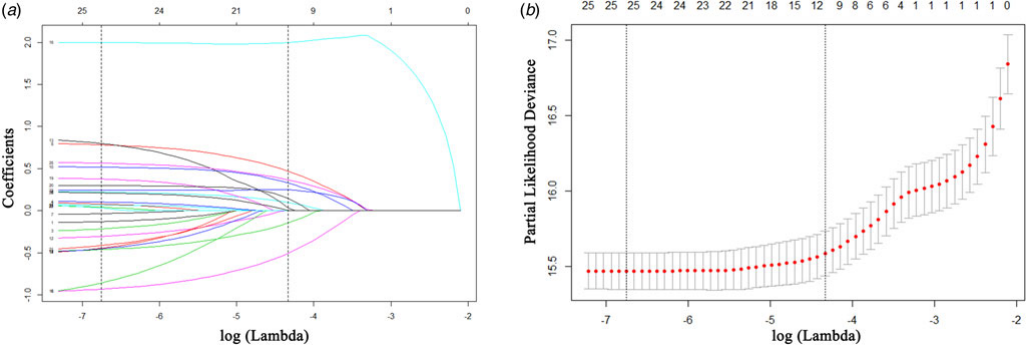
Predictor selection using the least absolute shrinkage and selection operator (LASSO). (a) LASSO coefficient profile plot of 26 features for survival. (b) Parameter (lambda) selection in the LASSO model adopted 10-fold cross-validation by the minimum criteria for survival.

In the training cohort, a total of 26 high-dimensional characteristics of HIV/AIDS inpatients were incorporated in the LASSO regression model. Then, a coefficient profile plot (Fig. 2a) and a 10-fold cross-validation plot (Fig. 2b) were constructed. To obtain the most regularised and efficient model, we selected 1 standard error of the minimum (1−SE criteria) by the cross-validated error plot. Finally, 11 variables were identified as potential predictors, including occupation, ART, pneumonia, tuberculosis, *Talaromyces marneffei*, hypertension, septicemia, anaemia, respiratory failure, hypoproteinemia and electrolyte disturbances.

### Development and external validation of the nomogram

**Fig. 3.**
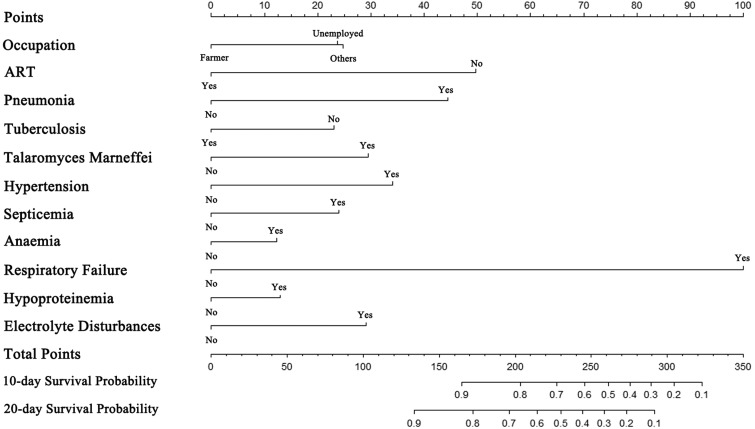
The nomogram for predicting the survival of hospitalised HIV/AIDS individuals.

Based on the primary data and actual clinical hospitalisation characteristics of the HIV/AIDS patients, we chose the 10-day and 20-day as the prediction time points of survival for the nomogram. The 11 selected predictors were all included in multivariate Cox regression modelling. The Likelihood χ^2^ statistic of the model was 516.30 (*P* = 0.000). Supplementary Figure 1 shows that the survival of inpatients with HIV was independently influenced by occupation (unemployed, *P* = 0.003; other non-farmer occupations, *P* < 0.001), ART before hospital admission (*P* < 0.001), pneumonia (*P* < 0.001), tuberculosis (*P* < 0.001), *Talaromyces marneffei* (*P* < 0.001), hypertension (*P* < 0.001), septicemia (*P* = 0.020), respiratory failure (*P* < 0.001) and electrolyte disturbances (*P* < 0.001). A simple-to-use nomogram was then developed, as shown in Figure 3.

**Fig. 4.**
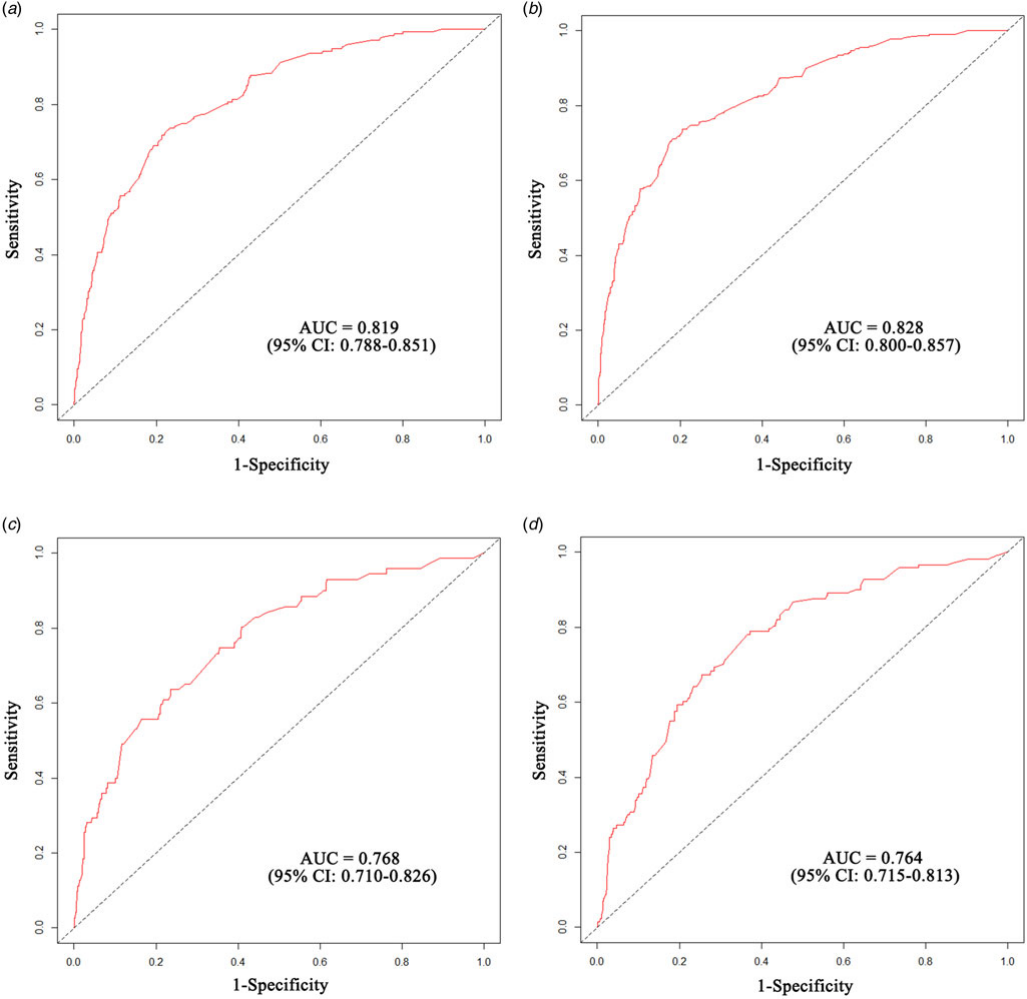
Receiver operating characteristic (ROC) curves of the nomogram model. (a, b). ROC curves plot of the training cohort for the 10-day and 20-day probabilities, respectively; (c, d) ROC curves plot of the external validation cohort for the 10-day and 20-day probabilities, respectively.

**Fig. 5.**
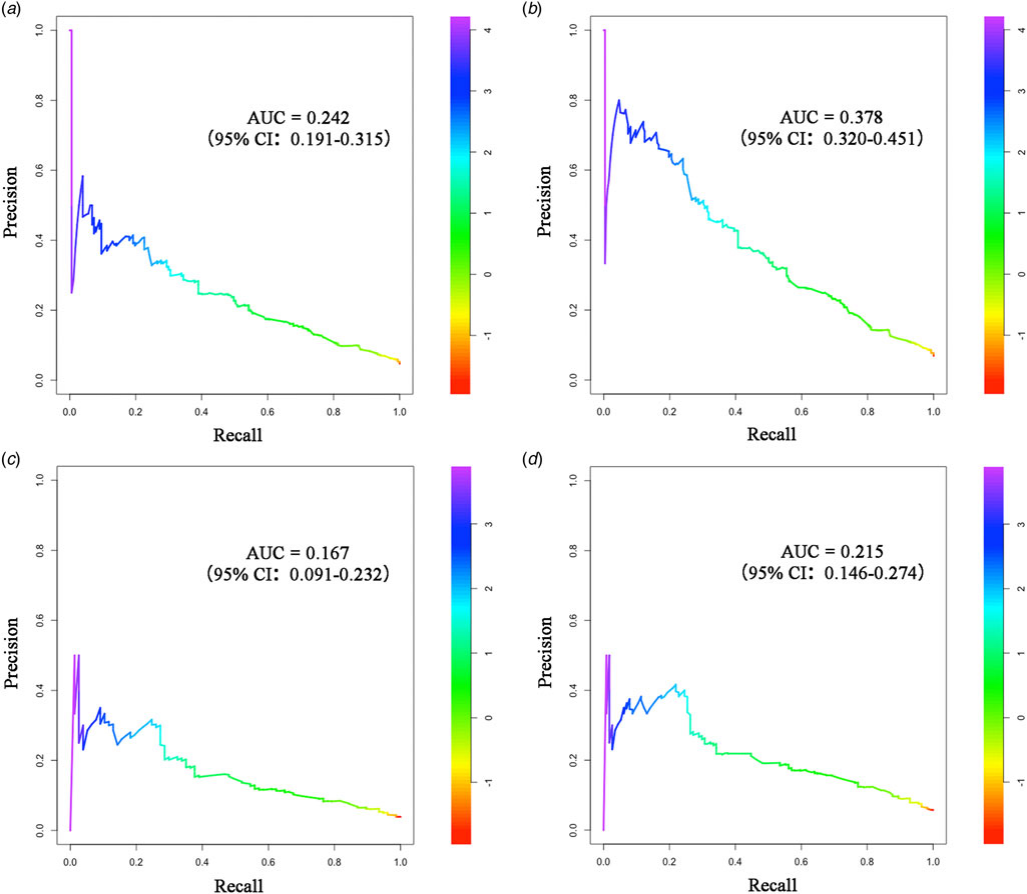
Precision-recall (PR) curves of the nomogram model. (a, b). PR curves plot of the training cohort for the 10-day and 20-day probabilities, respectively; (c, d). PR curves plot of the external validation cohort for 10-day and 20-day probabilities, respectively.

The nomogram was validated in a separate cohort of patients who were admitted between January and December of 2015. In the training cohort, the Integrated Brier Score was 0.076 (95% CI 0.061–0.101) and the Brier scores at 10-day and 20-day time points were 0.046 (95% CI 0.039–0.055) and 0.071 (0.063–0.080), respectively. In the external validation cohort, the Integrated Brier Score was 0.101 (95% CI 0.074–0.140) and the Brier scores of two time points were 0.041 (95% CI 0.032–0.050) and 0.077 (0.064–0.090), respectively. Therefore, the model has good discrimination for predicting 10- and 20-day survival among hospitalised PLWHA in this hospital setting. The AUC-ROC of nomogram in training cohort were 0.819 (95% CI 0.788–0.851) and 0.828 (95% CI 0.800–0.857) at two time points, respectively (Figs 4a and 4b). In external validation, the AUC-ROC were 0.768 (95% CI 0.710–0.826) and 0.764 (95% CI 0.715–0.813) at two time points, respectively (Figs 4c and 4d). The AUC-PR of nomogram in training cohort were 0.242 (95% CI 0.191–0.315) and 0.378 (95% CI 0.320–0.451) at two time points, respectively (Figs 5a and 5b). In external validation, the AUC-PR were 0.167 (95% CI 0.091–0.232) and 0.215 (95% CI 0.146–0.274) at two time points, respectively (Figs 5c and 5d).

### Calibration and decision curve analysis

**Fig. 6.**
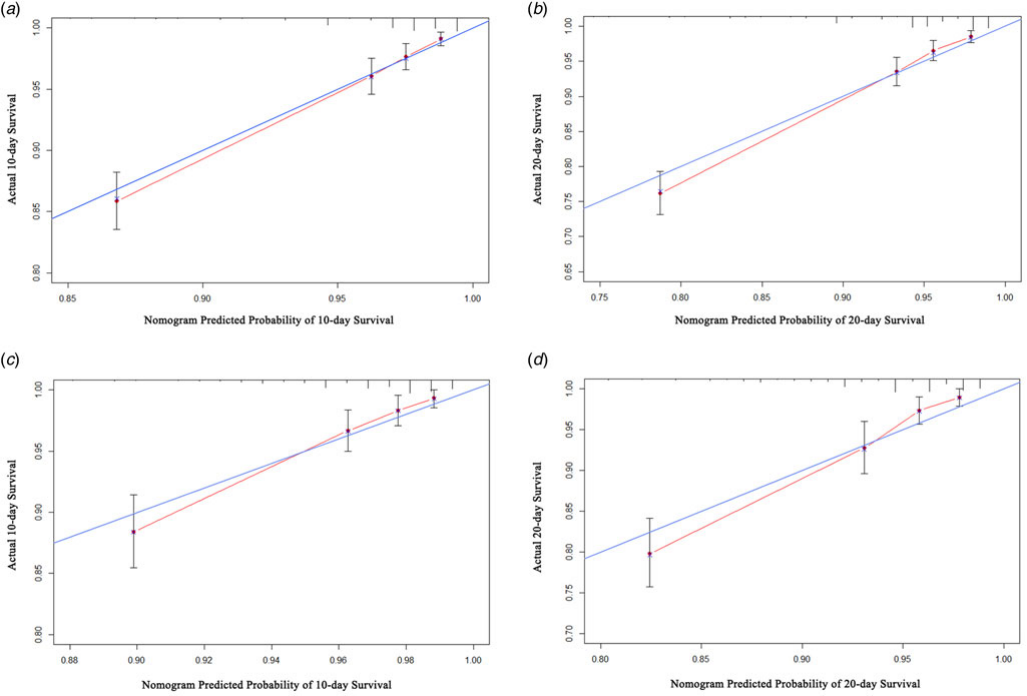
Calibration plots of the nomogram model. (a, b). Calibration plots of the training cohort for the 10-day and 20-day probabilities, respectively; (c, d). Calibration plots of the external validation cohort for the 10-day and 20-day probabilities, respectively.

Calibration plots revealed that the nomogram was well-calibrated in both the training (Figs 6a and 6b) and validation (Figs 6c and 6d) cohorts.

**Fig. 7.**
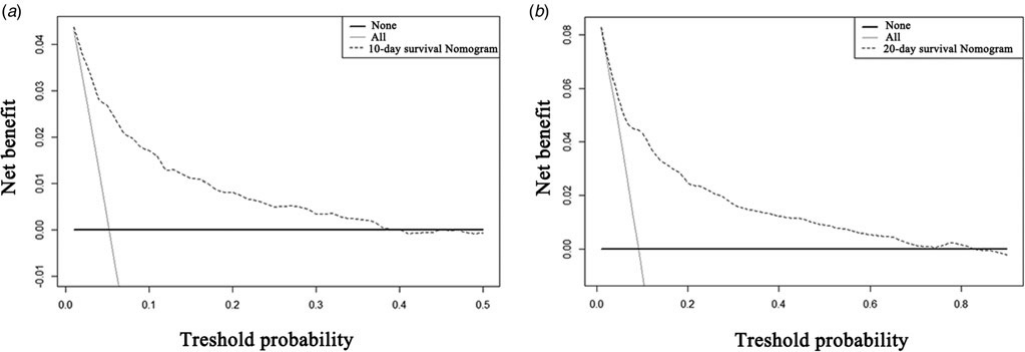
Decision curve analysis for the net benefit of the nomogram. The black solid line represents that none of the inpatients survived. The grey solid line represents that all of the inpatients survived. The black dashed line represents the model of the 10-day or 20-day survival nomogram in the training cohort. (a). Decision curve analysis for the nomogram predicting the 10-day survival; (b) Decision curve analysis for the nomogram predicting the 20-day survival.

The DCA for the nomogram was performed to evaluate its clinical utility in the training cohort (Figs 7a and 7b). When the threshold probability of an inpatient is 1% to 39% in predicting the 10-day survival or 1% to 82% in predicting the 20-day survival for an inpatient, the decision curve of the nomogram is higher than the two other extreme curves (the all inpatients died scheme and none of the inpatients died scheme). These results indicate that inpatient outcomes benefit more in the above range of the threshold probabilities and that the nomogram can assist in decision-making.

## Discussion

In this study, we developed and validated a simple visual nomogram as a new approach to predict the individual survival of HIV/AIDS patients for the first 10 and 20 days in the hospital. The nomogram we presented based on this hospital has relatively high predictive power and accuracy, with the Integrated Brier Score was 0.076 (95% CI 0.061–0.101) or AUC-ROC of 0.819 (95% CI0.788–0.851) and 0.828 (95% CI 0.800–0.857) at two time points. Low AUC-PRs were also found in this study, which may be due to the high-class imbalance of positive and negative samples. This particular condition is common in medical predictive model studies [22–24]. Furthermore, the validation of the nomogram supported its good predictive power and accuracy in predicting the survival of HIV-infected inpatients. Good calibration plots in the external validation cohort also confirmed the stability of the nomogram.

Although the Brier score has often been used to evaluate the predictive accuracy of a predictive model, it cannot indicate whether the model was clinically useful. In recent years, DCA has been used for assessing the clinical utility of a predictive nomogram and selecting the optimal model [25, 26]. Moreover, DCA can visualise the clinical net benefit of a predictive model at the different threshold probabilities [27]. According to the DCA in the current study, the developed nomogram is clinically useful for predicting the survival of inpatients with HIV/AIDS.

To screen the optimised predictors, the LASSO regression method was utilised in this study. LASSO is generally regarded to be suitable for the data dimension reduction of high-dimension clinical factors [28] and superior to the methods based on univariate differences in the variable selection for a predictive model [25]. It is also considered to enhance accuracy and interpretability [29, 30]. In our study, the nomogram incorporated 11 independent risk factors from 28 variables based on LASSO regression. The predictors not only covered the demographic characteristics and HIV-related complications but also included HIV-unrelated complications that may contribute to the death of inpatients. However, the limitations of the LASSO method should also be recognised. Firstly, the number of variables selected by LASSO could not exceed the number of samples. Secondly, LASSO could only select one or a few highly correlated variables.

Among the 11 predictors incorporated into the nomogram, most of them have been identified as risk factors for the death of HIV/AIDS patients in previous studies [2, 3]. Nevertheless, the unemployed and other non-farmer occupation variables in this nomogram are higher risk factors than the farmer variable. This finding is inconsistent with the results of a previous report showing that farmers accounted for 68.40% of new HIV-1 infections in Guangxi and had a higher mortality [31]. One possible explanation for this discrepancy is that rural areas and farmers in China including Guangxi have become a focus of HIV/AIDS preventive interventions in recent years [32, 33]. Various interventions such as the ‘County-Township-Village’ allied HIV prevention and control intervention in rural areas have greatly improved the knowledge and attitude of rural residents regarding HIV/AIDS [33]; thus, the hospitalised farmers in our study may have had a higher level of knowledge and greater willingness to receive ART. In addition, in our nomogram, tuberculosis is a beneficial factor for the survival of HIV/AIDS patients, which is contrary to the fact that tuberculosis is the leading factor for the death of HIV/AIDS patients. Two explanations may address this issue. First, it is well known that mycobacterium tuberculosis (Mtb) coinfection is one of the leading causes of death in HIV-positive patients [34, 35]. Thus, Mtb infection received more attention and effective intervention in the early stage of disease was adopted. These measures may lead to improved survival in the short stay during hospitalisation. In this study, the average survival time of hospitalised PLWHAs with Mtb (22.89 days) was longer than those without Mtb (15.12 days). Second, our study focused on the survival of patients with HIV during their hospitalisation; thus, the long-term effect of Mtb infection was not taken into account.

The management of HIV-infected inpatients in Guangxi is still a challenge for local AIDS prevention and control [15]. We expect our exploration to provide an innovative strategy to identify hospitalised PLWHAs at high risk of early death for HIV care and timely interventions in the clinic. The model is pragmatic for clinical doctors. Varying degrees of outcomes of inpatients could be identified quickly and expediently. Risk-adapted therapeutic strategies for inpatients can then be conducted in a timely manner to reduce or prevent death. Ideally, this algorithm can also be easily adapted to the internet or electronic case system for patients or clinicians and an automated tool would help timely decision-making and risk management. Moreover, the predictive nomogram can also help avoid excessive intervention and longer hospital stays of those with good outcomes.

However, this study has several limitations needed to be pointed out. First, this retrospective study was conducted in a single hospital in Guangxi, which may lead to selection bias and affect the extrapolation of the model. Nevertheless, the Fourth People's Hospital of Nanning (Guangxi, China) is the largest HIV/AIDS treatment unit in Guangxi. The inpatients came from all over Guangxi and even neighbouring provinces. In addition, the sample size of this study is rather large, which at least guaranteed authenticity among HIV/AIDS patients in Guangxi. Second, individualised intervention or detailed treatment information of HIV/AIDS inpatients during the hospital was not incorporated into the model due to incomplete data, which may weaken, to some extent, the clinical significance of the model. Finally, the clinical utility of the nomogram was not assessed in clinical practice in this study. Therefore, in the next step we expect to incorporate multicenter data and additional related clinical variables to develop a more effective and accurate nomogram for clinical use.

In conclusion, we developed a simple-to-use nomogram to predict the in-hospital survival of HIV/AIDS patients in Guangxi, China based on multiple aspects of patient information. This model had a relatively high performance after external-validation and was clinically useful according to the results of DCA.

## Supplementary material

For supplementary material accompanying this paper visit https://doi.org/10.1017/S0950268820000758. click here to view supplementary material
