# Supplementary figures and images for: Development and external-validation of a nomogram for predicting the survival of hospitalised HIV/AIDS patients based on a large study cohort in western China
Source: Epidemiol Infect. 2020 Apr 1;148:e84. doi: 10.1017/S0950268820000758 (PMC7189350; doi:10.1017/S0950268820000758)

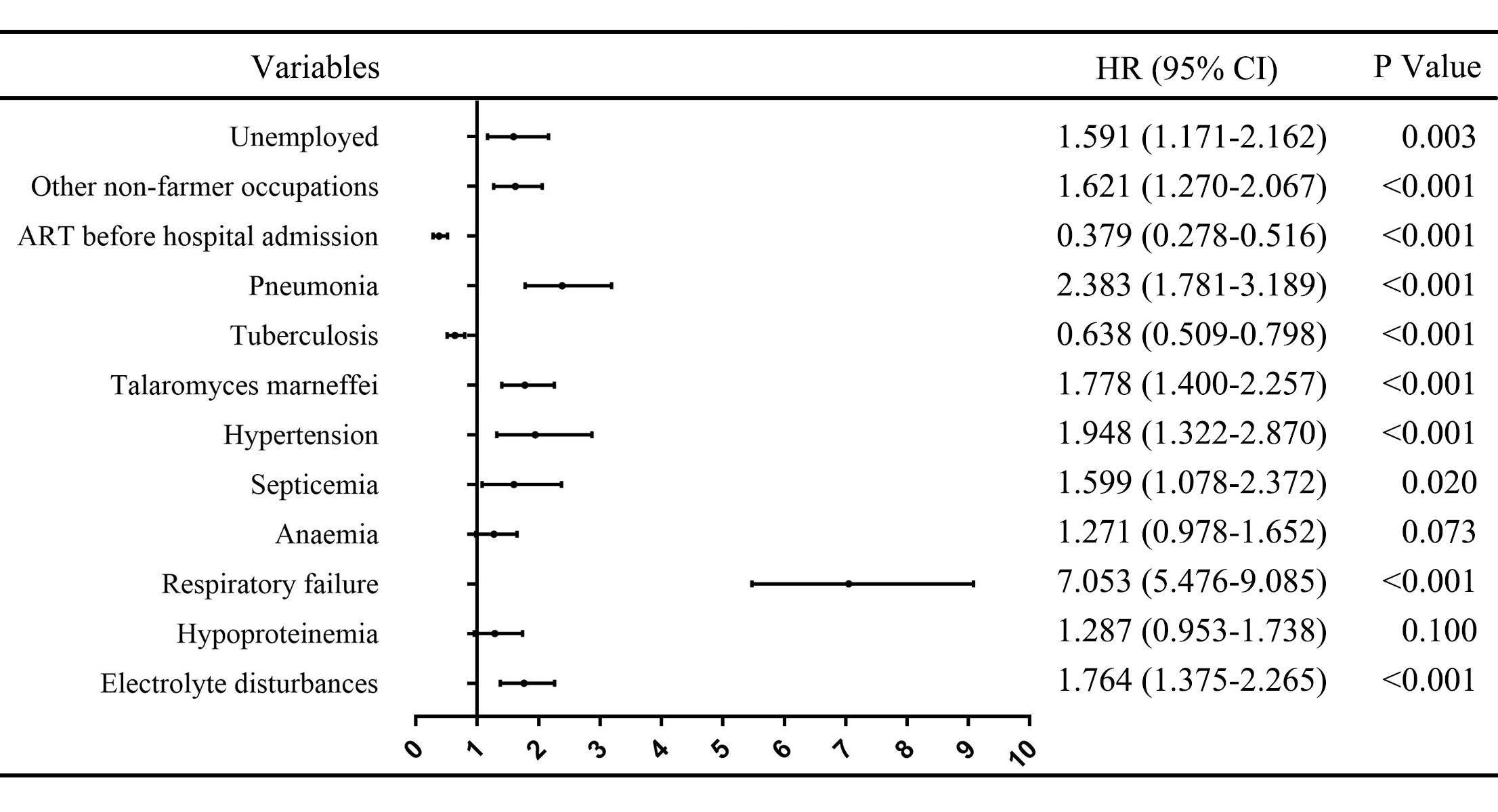

Supplement: Supplementary file 1 [file S0950268820000758sup001.tif]
